# Editorial: Atrial Fibrillation (AF) in heart failure with preserved ejection fraction (HFpEF)

**DOI:** 10.3389/fphys.2026.1897757

**Published:** 2026-06-25

**Authors:** Kirill Buturlin, Poojita Shivamurthy, Sotirios Nedios

**Affiliations:** 1 Department of Electrophysiology, Heart Center Leipzig, University of Leipzig, Leipzig, DE, Germany; 2 Cardiovascular Division, Perelman School of Medicine at the University of Pennsylvania, Philadelphia, PA, United States

**Keywords:** atrial fibrillation, catheter ablation, heart failure, HFpEF - heart failure with preserved ejection fraction, review, sex differences

## Introduction

The coexistence of atrial fibrillation (AF) and heart failure with preserved ejection fraction (HFpEF) is a growing epidemic that challenges our current therapeutic strategies. For too long, we have viewed them as separate entities. However, emerging evidence suggests they often arise from a shared pathophysiological substrate characterized by systemic inflammation, atrial and ventricular remodeling, impaired compliance, and elevated filling pressures.

In this context, AF is far more than an electrical trigger for hemodynamic collapse; it is a clinical manifestation of an underlying atrial cardiomyopathy. Managing these patients effectively requires us to look past rhythm control and address the issue of structural remodeling.

This Research Topic gathers diverse perspectives – from deep mechanistic insights to pragmatic clinical trials, underscoring that successful management of AF in HFpEF requires more than rhythm control alone.

## Beyond electrical instability: the cellular reality of remodeling

The study by He et al. offers a deep dive into why AF becomes increasingly difficult to manage as it progresses. Using single-cell analysis, the authors show a fundamental shift in atrial landscape with progression of AF. They observed a massive expansion of fibroblasts and leukocytes at the expense of healthy endothelial cells. What is particularly concerning for the clinician is the “plasticity” of embryonic fibroblasts, which seem to transform into aggressive, profibrotic phenotypes.

Essentially, these findings highlight a decline in protective signaling (like NPPA-NPR1) and an explosion of pro-fibrotic pathways. This contributes to the “stiff atrium” we often encounter in patients with HFpEF. The above findings may contribute to challenges we face in rhythm control, the frequent recurrence of AF despite ablation or anti-arrhythmic therapy in persistent AF and potentially why some patients experience minimal clinical improvement even after restoration of sinus rhythm.

## Rhythm control without consistent clinical benefit

The clinical data of a systematic review by Chen et al. evaluated the effect of catheter ablation in HFpEF patients in rhythm outcomes and hard clinical endpoints. While catheter ablation significantly improved freedom from AF compared to medical therapy, it failed to reduce heart failure hospitalization or mortality. Interestingly, when compared to HFrEF patients, those with HFpEF showed similar rhythm efficacy, but less consistent hard clinical benefits. These sobering findings point out that the underlying substrate in HFpEF is a different problem altogether.

Similarly, different mechanisms have been identified in women, who usually have worse and often atypical symptoms and lower quality of life (Odening et al., 2019). This was confirmed in a study by Zylla et al. that examined women with HFpEF undergoing cryoballoon ablation. Despite high procedural success and low complication rates, these patients had more AF-related rehospitalizations, numerically more recurrences, persistent biomarker abnormalities, and, most importantly, no real improvement in quality of life. These findings suggest that for this subgroup, AF is often a part of a systemic HFpEF process.

Ultimately, these studies confirm that in the HFpEF population, rhythm improvement and clinical recovery are not always synonymous. This discrepancy likely reflects the fact that AF in HFpEF often develops on top of an established atrial and ventricular myopathy.

## Phenotype and disease stage matter

The importance of advanced remodeling, beyond rhythm control, is also shown in the study by Liu et al. who examined functional tricuspid regurgitation (FTR) in patients with persistent AF undergoing catheter ablation. In their cohort, significant FTR – particularly the ventricular phenotype – was a strong predictor of poor outcomes and lower ablation success. Notably, while successful rhythm control often led to an improvement in TR severity, AF recurrence almost guaranteed that the valve disease would persist or worsen. Thus, FTR is not simply a bystander, but an indicator of more advanced remodeling.

Even though this study wasn’t limited to HFpEF, its implications are direct. AF progression is inseparable from global structural remodeling. In many cases, late-stage bi-atrial disease or right-sided involvement may represent a “point of no return” where ablation is less effective. For the HFpEF population, where right heart congestion and pulmonary pressures often dictate the overall outcome, identifying these phenotypes early is essential.

## Toward phenotype-tailored and substrate-adapted ablation

Parwani et al. discuss the emerging role of pulsed field ablation (PFA) for AF patients with HFpEF. In this specific cohort, the goal of ablation likely extends beyond simple rhythm control to the preservation of atrial mechanics. Since PFA is non-thermal, it may limit collateral injury, fibrosis, and impairment of atrial compliance compared to conventional thermal approaches (cryoablation and radiofrequency) – a critical advantage in HFpEF, where diastolic filling is highly dependent on atrial function. However, as the authors note, evidence remains preliminary and HFpEF’s phenotypic heterogeneity mean that further exploration of PFA in this patient subset is essential.

The need for tailored strategies is further illustrated by Fitouchi et al. in a complex case of transthyretin cardiac amyloidosis. In a setting of diffuse low-voltage zones and perimitral flutter, ethanol infusion into the vein of Marschall was necessary to achieve a durable mitral isthmus block and further long-term arrhythmia control and clinical improvement. While a single case report cannot define practice, it highlights an important principle: in patients with severely remodeled atria, substrate-adapted approaches may be necessary to achieve durable procedural success.

## Conclusion

In summary, the studies in this Research Topic reinforce that AF in HFpEF is not merely an electrical problem, but a manifestation of progressive atrial cardiomyopathy, characterized by fibrosis, endothelial dysfunction, inflammatory signaling, and progressive chamber remodeling. While catheter ablation remains a powerful tool for rhythm control, its clinical success is increasingly defined by the timing of intervention and the baseline structural remodeling. Future considerations include early recognition of atrial myopathy with advanced imaging and biomarkers, aggressive treatment of underlying risk factors, therapeutics for halting adverse remodeling combined with early AF detection and timely arrhythmia intervention. Altogether, these contributions move the field toward a more personalized approach to AF in HFpEF.

## References

[B1] Odening K. E. Deiss S. Dilling-Boer D. Didenko M. Eriksson U. Nedios S. . (2019). Mechanisms of sex differences in atrial fibrillation: role of hormones and differences in electrophysiology, structure, function, and remodelling. Europace 21, 366–376. doi: 10.1093/europace/euy215 30351414

